# Exploration of the barriers and facilitators influencing use of telehealth for orthotic/prosthetic services in the United States of America: An orthotist/prosthetists perspective

**DOI:** 10.1371/journal.pone.0309194

**Published:** 2024-10-24

**Authors:** Michael Dillon, Emily Ridgewell, Leigh Clarke, Katie Bishop, Saravana Kumar

**Affiliations:** 1 Department of Physiotherapy, Podiatry, Prosthetics and Orthotics, School of Allied Health, Human Services and Sport, Health Sciences, La Trobe University, Melbourne, Victoria, Australia; 2 IIMPACT in Health, Allied Health & Human Performance, University of South Australia, Adelaide, South Australia, Australia; Iran University of Medical Sciences, ISLAMIC REPUBLIC OF IRAN

## Abstract

Innovative models of healthcare, such as telehealth, are required to meet the growing demand for orthotic/prosthetic (O&P) services. While O&P users report being very satisfied with telehealth, many clinical facilities have reverted to in-person modes of care as COVID-19 restrictions have eased. As such, there is a disconnect between benefits of telehealth to O&P users, and the clinical services being delivered in-person. The aim of this study was to explore the orthotist/prosthetist’s perspective of the barriers and facilitators influencing use of telehealth in the United States of America (USA). O&P practitioners were recruited from across the USA. In-depth, semi-structured interviews were used to document practitioner demographics, the services being provided using telehealth, and practitioners’ perspective of the barriers and facilitators influencing use of telehealth. Data describing participant demographics and services were summarised. Interviews were transcribed verbatim and analysed using thematic analysis. 30 practitioners from across the USA participated. Telehealth was used to deliver a range of O&P services including: initial evaluations, routine follow-ups, and delivery of a device in rare circumstances. Barriers to using telehealth included: poor phone/internet connection and lack of access to technology. Facilitators to using telehealth included: a patient-focussed attitude, and recognition of the benefit of telehealth. Telehealth is being used across the entire spectrum of O&P care. Once the significant barriers were resolved, like access to reliable internet/phone reception, telehealth was feasible. An outstanding telehealth experience was facilitated by practitioners who focused on the benefits that telehealth can provide (not the limitations), as well as giving O&P users agency over the choice to use telehealth. There are opportunities to improve access to safe and effective O&P telehealth services by adopting a right-touch approach to practitioner regulation, and advocating for reimbursement that supports better systems and procedures within clinical facilities.

## Introduction

The number of people who require orthotic/prosthetic (O&P) services is expected to double by 2050 [1]. It will be difficult for the O&P workforce to meet this growing demand given the number of practitioners per head of population, and the geographic clustering of clinical services in metropolitan areas and large regional centers [2–7]. Innovative models of service delivery, such as telehealth, may improve efficiency and address inequality in access; particularly for those living in regional and remote areas where the greatest health inequalities exists [8].

Telehealth has been defined as the delivery of healthcare where the patient and healthcare professional are remote from one another [9]. This definition extends beyond different forms of telecommunication consistent with recent evidence about the ways that contemporary telehealth—including photo sharing and courier/postal services—has been used to deliver O&P services. [10]

Recent research highlights that telehealth offers many benefits such as convenience and efficiency given reduced travel time [11–13]. Patients are typically very satisfied with the care they received using telehealth [14, 15]. Moreover, telehealth is equally or more effective when compared to in-person care, across a range of health-related disciplines [16].

While the evidence for telehealth is positive, it is also discipline-, application- and context- specific [16], with little evidence of telehealth applications in O&P. Early literature in O&P described the use of telehealth for discrete technical applications such as remote configuration of prosthetic devices [17–19] or to connect O&P practitioners with clinical specialists (e.g., a scoliosis specialist) [20–22]. A second wave of literature emerged more recently given many O&P services had adopted telehealth to continue essential healthcare during the COVID-19 pandemic. This literature described the impact of telehealth on O&P service delivery during the COVID-19 pandemic [23, 24] or the patient perspective of O&P services provided via telehealth [10]. The latter of these studies [10] also described a range of barriers (e.g., lack of agency and control over the decision to use telehealth) and facilitators (e.g., a well-established working relationship between patient and practitioner) influencing the use of telehealth from the perspective of O&P users.

While recent research has given us some insight into the barriers and facilitators influencing the use of telehealth from the perspective of O&P users, there is no research describing the perspective of O&P practitioners despite a range of issues that likely impact the willingness of a practitioner or service to offer telehealth including: limited reimbursement [25], complex regulations that differs between jurisdictions [26], and guidelines developed rapidly during the COVID-19 pandemic based on the limited evidence available [23, 25, 27–32].

If we are to improve efficiency and access to safe and effective O&P care, it is important that we understand the practitioners’ perspective of the barriers and facilitators that influence the use of telehealth services. What we learn through this research may help to identify some of the barriers that limit the uptake of telehealth, as well as some of the best-practice examples that facilitate provision of safe and effective O&P services.

We chose to conduct this study in the USA given the large number of certified orthotist and/or prosthetists that may help minimise the recruitment challenges reported in recent telehealth research [10].

Given this background, the aim of this study was to explore the orthotist and/or prosthetists’ perspective of the barriers and facilitators influencing use of telehealth in the United States of America (USA).

## Materials and methods

Ethics approval for this study was granted by the Human Research Ethics Committees at La Trobe University (HEC#21405) and the University of South Australia (HREC# 204480).

### Participants

Participants met the following inclusion criteria: certified orthotists and/or prosthetists (hereafter referred to as practitioners), adults > 18 years of age, and able to participate in an English-language interview. We were deliberate in not requiring participants to have experience using telehealth given the perspectives of people who do little or no telehealth is valuable in identifying barriers and facilitators.

Practitioners were convenience sampled through the *American Board for Certification of Orthotics*, *Prosthetics and Pedorthics* (ABC) via email invitation to their membership database, as well as promotion through their e-bulletin and website. Practitioners were also purposively sampled using a maximum variation strategy. That strategy sought to ensure the diversity of the sample by deliberately targeting the recruitment of practitioners with specific experiences (e.g., those who work in small clinical facilities). For both sampling strategies, one reminder email was sent one-week after the initial invitation.

### Procedure

For each practitioner who responded, an investigator (ER) recorded their contact details, confirmed eligibility, scheduled an interview, provided a video-conferencing link (Zoom, San Jose California, USA) and a copy of the Participant Information Statement. One reminder email was sent in the week prior to the interview.

Once the target number of interviews were scheduled (n = 30), additional practitioners were waitlisted knowing they may be contacted as part of the planned maximum variation sampling strategy, or to reach data saturation.

Of the 30 practitioners with a scheduled interview, five did not participate. As such, following the first 25 interviews, investigators reviewed the demographics of the sample and determined that greater representation was required from practitioners who had provided telehealth services through Veterans Affairs, or while working in the southern states of the USA. An investigator (ER) emailed practitioners on the waiting list who had worked in these settings. Five practitioners who had worked in these settings responded to this invitation. Interviews for these five practitioners were scheduled following the previously described procedure.

### Data collection

One-on-one, semi-structured interviews were conducted in accord with an interview guide (S1 File) that was developed, drafted and refined according to best-practice guidelines [33, 34].

The interview commenced with the practitioner describing the purpose of the study, reaffirming their eligibility, and asking any questions prior to verbally consenting. Practitioners then answered demographic questions (e.g., sex, age, clinical certification in orthotics or prosthetics) and provided details about the clinical service where they worked (e.g., large nation-wide service provider). The body of the interview included open-ended questions with nested prompts to facilitate a rich conversation (S1 File).

Each interview was audio-recorded and lasted approximately 45 minutes. Interviews were conducted by a female investigator (ER) with undergraduate training in O&P, clinical experience working in paediatric orthotic services, and post-graduate doctoral training. The investigator (ER) completed a reflective diary following each interview to become more aware of feelings, assumptions, personal biases, and decisions [35].

### Data analysis

Demographic data were synthesised using descriptive statistics appropriate to the data type.

An epidemiological map describing the spread of practitioners was developed using the R language environment (Vienna, Australia); specifically, the Simple Features for R and ggplot packages [36–38]. Population data were taken from the cartographic boundary file from the 2018 US census [39]. Geographic location of participants was determined using primary service location reported in the interview, from which precise coordinators of latitude and longitude were obtained using Google Maps [40].

Data were analysed using a range of strategies to engender confidence in the rigour and trustworthiness of the analysis [41]. Audio recorded interviews were transcribed verbatim by a professional transcription service (REV transcription, San Francisco, CA) and uploaded to NVivo 11 (QSR International, Burlington, MA). All transcripts were independently read and analysed by the interviewing investigator (ER) and one co-investigator (MD). Using an inductive approach, each transcript was independently coded line-by-line. Subthemes were extracted from categorizing the codes, and themes were extracted from categorising the subthemes. Investigators (ER,MD) attended reconciliation meetings where they discussed their differing coding and interpretations to reach consensus, considered whether any new interpretations or themes emerged, and identified topics that should be followed up in subsequent interviews. This allowed investigators to monitor whether data saturation had been reached given the target sample. As part of this member-checking process, practitioners were sent a 1–2 page summary of their interview including themes, illustrative first-person quotes, and any queries that arose during the coding and reconciliation. Practitioners were invited to provide feedback via email or phone. Amendments were made to ensure the themes reflected the practitioners’ perspective. Data analysis continued in this way until data were available for 30 practitioners, and we were confident that data saturation had been reached.

To synthesise data across participants, investigators (ER,MD) independently identified common themes and subthemes using a first principles approach. Differences in the interpretation were discussed at regular meetings until consensus was reached. The narrative for each theme and subtheme were drafted and illustrative first-person quotes selected (ER,MD).

To engender confidence in the interpretation, each theme and subtheme was presented to members of the research team (ER,MD,SK,LC) in a half-day meeting. Queries were cross-checked against the original transcription and the reconciled coding to inform changes to the results narrative. To triangulate the data and minimise the risk of confirmation bias, an investigator (KB) who had not been involved in the interviews, coding, write up, or discussion about results, reviewed each interview transcript in a random order and summarised the main themes. These themes were cross-checked against the themes in the original member checking documents. After reviewing all interviews, the investigator (KB) compared the results narrative with their own interpretation. Queries were cross-checked against the original transcription and coding to inform changes to the results narrative.

To protect the privacy of participants, their real names have been replaced with pseudonyms. Similarly, identifying demographic information (e.g., place of employment) has been omitted.

## Results

### Participant demographics

On average, practitioners were middle aged (47±14 years), dual certified as orthotists and prosthetists (80%), with many years post-certification experience (20±13 years), and currently worked in multi-site private- (67%) or publicly-listed companies (20%, Table 1). Practitioners spent most of their time in clinical activities (74±30%) and served both adult and paediatric patients (87%, Table 1).

**Table 1:** Participant demographics.

**Sex (n, %)**		
	Male	16 (53%)
	Female	14 (47%)
**Age (years)**		
	Mean	47.2
	Standard deviation	13.5
	Range	27–73
**Certification (n,%)**		
	Both orthotics & prosthetics	24 (80%)
	Orthotics only	4 (13%)
	Prosthetics only	2 (7%)
**Years since certification (years)** *		
	Mean	19.8
	Standard deviation	12.9
	Range	1–39
**Disciplines of clinical practice (n, %)**		
	Both orthotics & prosthetics	26 (87%)
	Orthotics only	3 (10%)
	Prosthetics only	1 (3%)
**Self-described clinical speciality/s (n, %)** ^#^		
	Paediatrics (non-specific)	9 (30%)
	Generalist	7 (23%)
	Lower limb prosthetics	5 (17%)
	Upper limb prosthetics	5 (17%)
	Geriatrics	5 (17%)
	Paediatrics (cranial)	4 (13%)
	Paediatrics (scoliosis)	3 (10%)
	Compliance or auditing	2 (7%)
	Neuro orthotics	1 (3%)
	Sports medicine	1 (3%)
**Self-described role (n, %)** ^#^		
	Clinician	26 (87%)
	Management (e.g., clinic or business manager)	11 (37%)
	Educator or trainer	5 (17%)
	Owner	4 (13%)
	Travelling specialist	3 (10%)
	Compliance or auditing	2 (7%)
	Research or project activities	2 (7%)
**Estimated proportion of time dedicated to clinical activities (%)**		
	Mean	73.5
	Standard deviation	29.8
	Range	0^^^-100
**Type of organisation**		
	Privately owned clinic	20 (67%)
	Publicly owned clinic	6 (20%)
	Hospital/Not-For-Profit	3 (10%)
	Other	1 (3%)
**Number of clinical sites within organisation**		
	Single	6 (30%)
	Multi-site	24 (80%)
**Number of certified practitioners employed at organisation**		
	Mean	7
	Standard deviation	6
	Range	1–32

*when participants became certified in orthotics or prosthetics at different times, the number of years since their earliest certification was recorded.

^three participants were not practicing clinically at the time of this study. These participants described their most recent clinical role and speciality.

^#^participants often described more than one clinical speciality or type of role.

**Fig 1:**
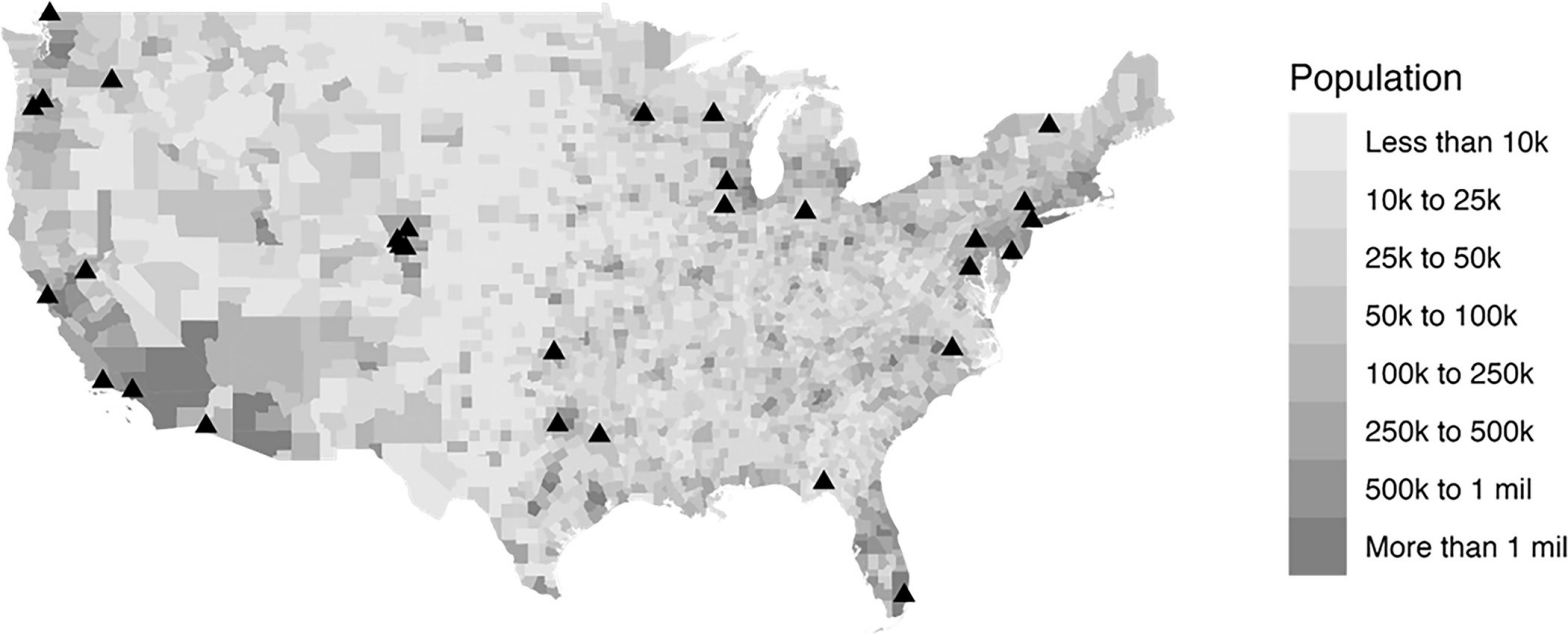
Practitioner location (black triangle) and US population density [39] at the county level (shaded areas).

While practitioners were geographically spread across the USA, they tended to be clustered in large population centres such as Chicago in the Midwest (Fig 1).

### Modes of telehealth and the types of O&P services

**Fig 2:**
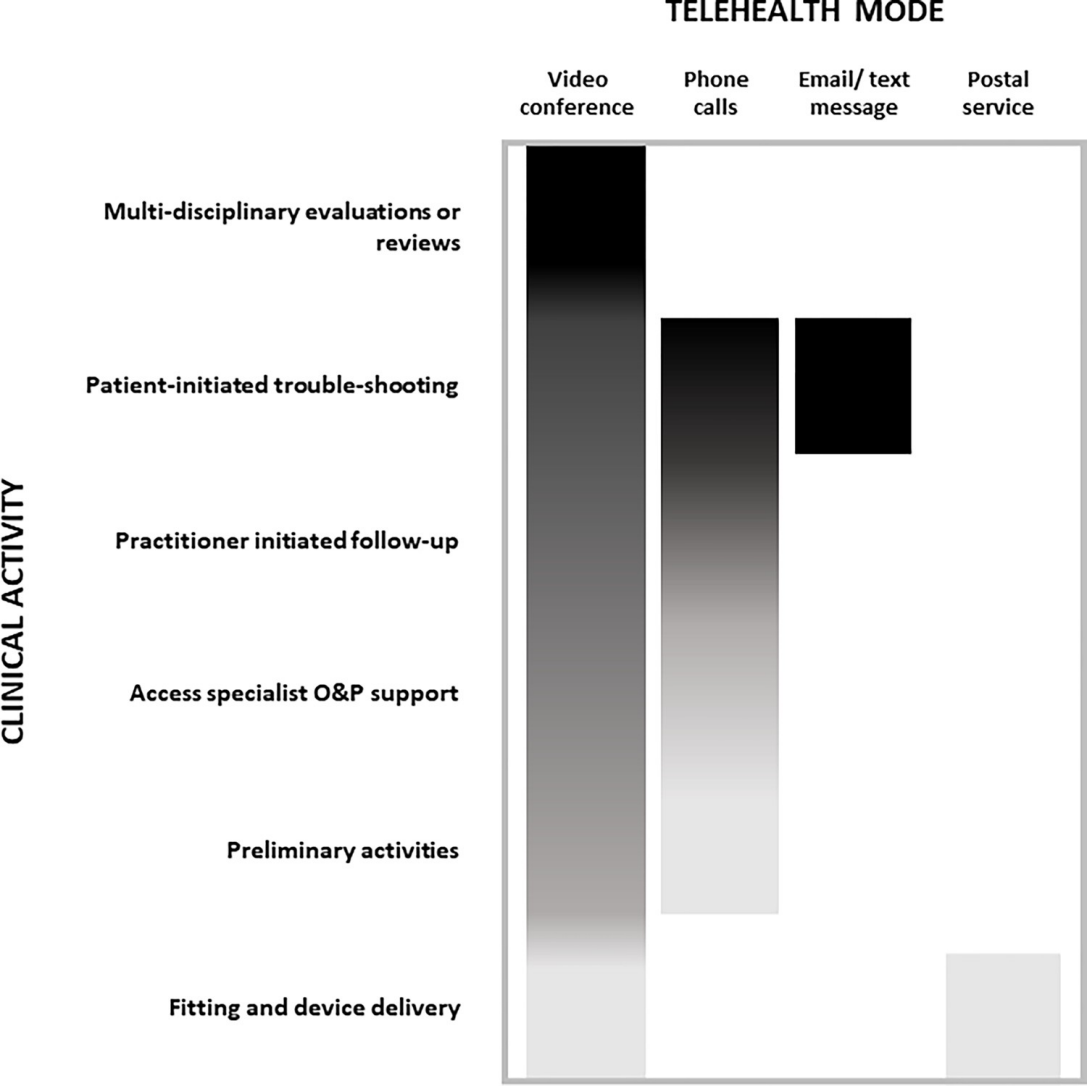
Frequency of clinical activity and telehealth mode. Darker colour indicates higher frequency.

The full spectrum of O&P services were provided using a variety of telehealth modes including: video conferencing, landline or cell calls, text message, email, and postal/delivery services (Fig 2).

Video conferencing was used across the full spectrum of O&P services; most often to connect multiple health practitioners (e.g., O&P practitioners, prescribing physician) in the presence of the O&P user to ensure alignment on the clinical treatment plan. To a lesser degree, video conferencing was used for initial consults or education (e.g., pre-amputation consultation), routine and scheduled follow-up appointments (e.g., check the fit of a cranial orthosis), or where a clinical specialist (e.g., an upper limb prosthetics specialist) was supporting a practitioner to provide direct patient care. In rare cases, video conferencing was used for remote delivery of an orthosis/prosthesis with justification (e.g., fitting a scoliosis orthosis where COVID-related restrictions prevented in-person appointments). In all cases, video conferencing was practitioner initiated.

*.. we’ve done … [video] conference calls to evaluate [i.e., initial assessment] whether someone may be able to benefit from an AFO [Ankle Foot Orthosis]… we know we’re going to have to see the patient, but the therapy department wants to participate in the conversation early on to make sure*… *that’ll benefit the patient. (Johan)*

*[The video conference]* …*was for a young child who was going to be undergoing an amputation*, *… and the parents wanted to learn more about prostheses*, *the whole process*… *(Lauren)*

By contrast, cell phone calls, texts, or emails (including photos) were most often used by O&P users to initiate contact with a practitioner to trouble-shoot issues. Where the practitioner considered the issues were serious, video conferencing was typically the second step to investigate. Practitioners also used phone calls, most often for ad-hoc follow-ups (e.g., non-attendance for a review appointment).

*The father [patient’s father] texted me*… . *“[Patient’s name] is having a problem with his brace, he says it’s digging into his shoulder blade. The pad is bothering him”. I said, “okay, I can do FaceTime with you.” (Maria)*

*… that usually starts off through email communication or a phone call [from the patient] and they want to know if they can show me something*. *So*, *we’ve done* … *[video conferencing] so that I can see what they’re talking about*. *(Charlie)*

Postal/delivery services were often used to send consumables to an O&P user (e.g., replacement prosthetic liner). By contrast, these services were rarely used to send a device directly to an O&P user (e.g., replacement orthoses that an O&P user is familiar with) or to a physical- or occupational- therapist for fitting with the remote support and oversight of the O&P practitioner.

*[It’s] just easy to send somebody some socks*… *or whatever they may need to get them going right then and there. (Reagan)*

*If they’re a regular [O&P] user*, *if they used the device for years*, *and we felt comfortable and the patient felt comfortable having something shipped directly to their home*, *then we’d do the fitting virtually*… *We were very selective in terms of the patients we identified to do that …*. *(Jackson)*

### Barriers and facilitators

**Fig 3:**
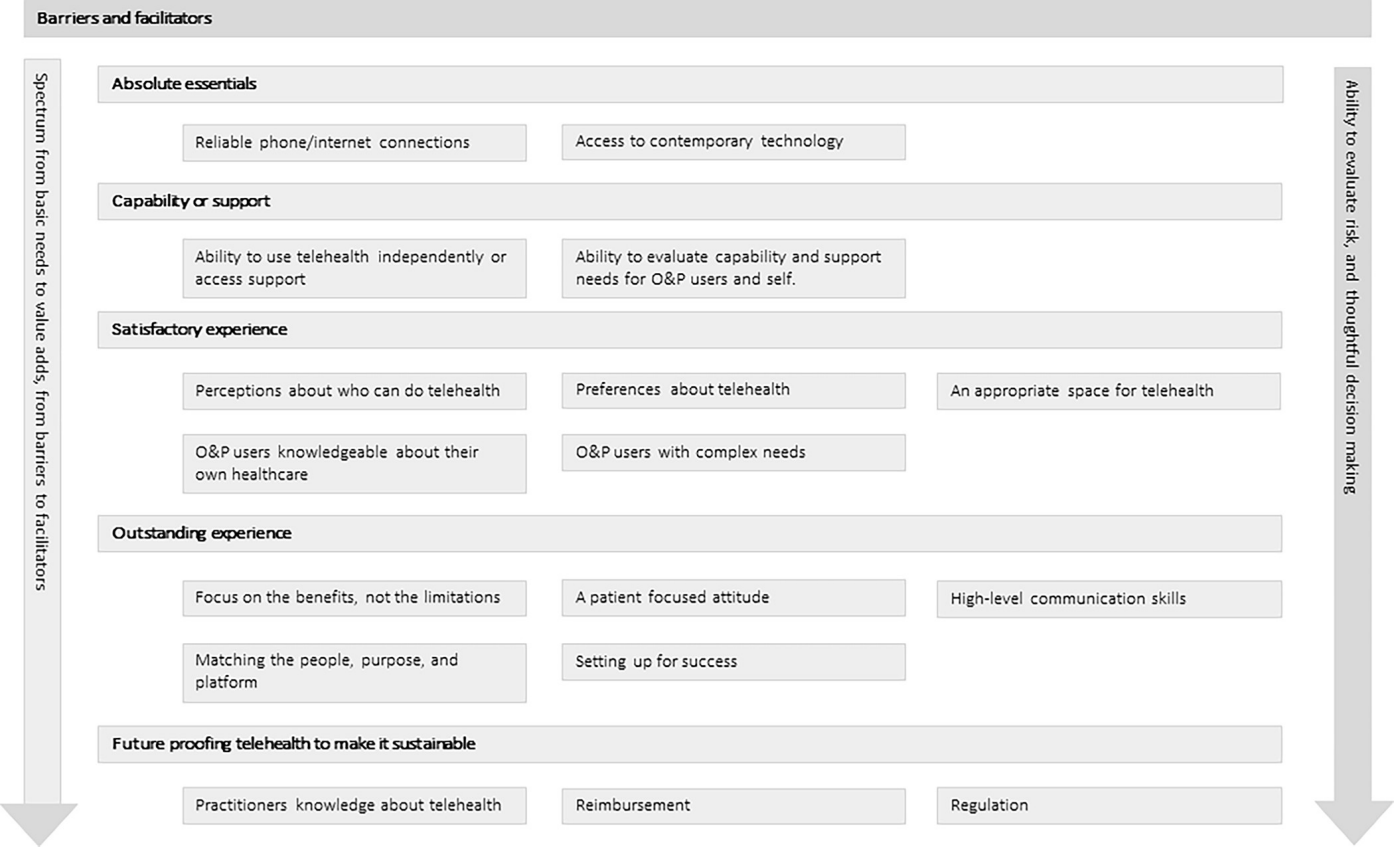
Barriers and facilitators to the use of telehealth to provide O&P services.

Participants described a wide variety of barriers and facilitators to the use of telehealth for O&P services. These may be considered along a continuum from absolute essentials that characterise the basic needs people have to participate in telehealth (e.g., reliable phone/internet connection), through to value adds that help create an outstanding telehealth experience (e.g., practitioners with high-level communication skills). Each of the themes and subthemes have been schematically presented to help readers navigate the detail of the results narrative (Fig 3).

#### Absolute essentials

To participate in telehealth, practitioners and O&P users need access to cellular/internet service *and* contemporary technology (e.g., computer or phone) that was easy to use and reliable.

*Reliable phone or internet connection*. While issues with cellular coverage tended to affect those living in rural areas, issues with poor internet were universal; even in metropolitan cities and large organisations such as tertiary hospitals.

… *poor internet connection at whatever site they’re at [patient], or if they’re in a more rural area, that video connection sometimes can be pretty bad. (Charlie)*

*Internet connection tends to be more difficult in hospitals*… .*[Hospitals] have more troubles with freezing*, *not dropping out*, *things like that*. *(Jackson)*

*Access to contemporary technology*. Such as a smartphone or a computer with a webcam, was a common barrier identified by practitioners that particularly seemed to impact O&P users. Practitioners described O&P users who did not have access to contemporary technology including people who: were older, lived in nursing facilities, lived on a fixed-income, or belonged to cultural or religious groups. For practitioners, the barriers related to software availability and whether it was installed, ready-to-use, and available to O&P users.

*For those [O&P users] that struggle with technology, half due to cognitive status, the other half just due to … lack of access to the technology, because a lot*… *are on … Medicaid programs or they’re on a fixed income. (Tess)*

#### Capability or support

*Ability to use telehealth independently or access to support*. Once the absolute essentials were in place, practitioners noted that both O&P users and practitioners needed the capability to utilise telehealth independently *or* access to supports to resolve technical challenges (e.g., audio or video doesn’t work).

*…as far as facilitating on the patient end,*… *it’s somebody to help with technology, whether it is a spouse, a child,… (William)*

*The barriers were the technical capabilities at the other end* [O&P user’s end]. … *a lot of time was spent trying to make microphones work*, *trying to make videos work*, *trying to get the sound to work*. *(David)*

*Ability to evaluate capability and support needs*. The ability of practitioners to evaluate an O&P user’s capability to use telehealth or ensure the necessary supports were available, was key to decisions about the feasibility of telehealth. Similarly, both O&P users and practitioners needed to self-evaluate their own capability, and request support as required (e.g., a practitioner seeking help to set up a software application).

*I think reading your patient really carefully is super important, because asking someone in their eighties to do a Zoom call with you may not be as successful as asking someone in their twenties to do a Zoom call with you*. *(Travis)*

#### Barriers to a satisfactory experience

Once the absolute essentials were in place, and both O&P users and practitioners had the capability or support they needed, telehealth was feasible. While telehealth may be feasible, there were still a range of barriers to a satisfactory telehealth experience.

*Perceptions about who can do telehealth*. There was a common perception among practitioners that some groups of O&P users were hesitant or less capable of using telehealth; in particular, older people. A few practitioners were able to look beyond age as an indicator of someone’s capability to use telehealth, and thoughtfully consider the individual and the supports available.

*I think it totally depends on the person. My grandfather is 94. He knows how to video chat with all of his friends*…*. He is Mr. Tech-savvy …. I [also] think that a lot of our patient population has cognitive delays and limited cognition due to early-onset dementia… They might have an iPhone, but they don’t know how to do anything besides answer a call (Tess)*

*Preferences about telehealth*. Practitioners recognised that their comfort with telehealth and preference for a particular telehealth mode (e.g., phone) determined what sort of telehealth was offered to O&P users. In the same way, practitioners reflected that O&P users often had their own preference for how they wanted to connect with practitioners. For example, some people felt uncomfortable on camera, others did not want to disclose an email address, or were anxious about using the technology.

*I’ve worked with a number of people [i.e*., *practitioners] who were very open about [the fact that] they don’t like it [i.e., video conferencing]. Those people who are not comfortable with technology are going to be more hesitant to use it. (William)*

*We have some patients that prefer email over phone and then I have some patients who refuse to give us emails [addresses]*. *(Eloise)*

*An appropriate space*: Practitioners noted that access to an appropriate space was a barrier to a satisfactory telehealth experience; particularly given the negative impact of background noise or distractions, or the size of the physical space and technology set up.

*Our practice is busy and loud and there’s machinery. Sometimes we’re sneaking into a closet or a room*… *to try to find a quiet space …. (Tess)*

*We ended up asking parents to do the [video conferencing] assessment in the patient’s bedroom*, … *because*… *their telehealth setup … would be in an office with a desk and it was difficult to … get enough space to see the patient*….*to have them walk more than three feet*. *(Jackson)*

*O&P users knowledge about their own healthcare*. Practitioners highlighted that some O&P users did not share the same technical language which made it difficult to describe the problems they were experiencing. For example, sometimes O&P users used different terms to describe parts of a prosthesis (e.g., liner, sleeve, or sock) which made it difficult for practitioners to understand the problem. Similarly, practitioners observed that some O&P users were unable to recognise that a problem existed, or they would describe the solution without understanding the cause of the problem.

…*there’s hundreds of examples of patients just substituting words they think fits …. [they] might say their liner, but what they mean is their … flexible inner socket. (Theo)*

*A patient said*, *"Yeah*, *I just need new liners*.*" I got new liners for him*. *He came in*. *He’s like*, *"Yeah*, *but my leg falls off randomly sometimes*.*" I’m like*, *“Oh*, *your lock mechanism is broken”*. *(Tess)*

*O&P users with complex needs*. Practitioners identified that a satisfactory telehealth experience was challenging when working with O&P users with complex needs such as older adults with communication difficulties (e.g., aphasia), hearing or vision loss, dementia, or where translation was required.

*…[for] the diabetic, dysvascular, elderly amputee that has visual impairments, hearing impairments, some cognitive or dimensional decline*…*, it [telehealth] becomes very challenging. (David)*

Some practitioners noted the challenges of a satisfactory telehealth experience where the objective evaluations were complex, or the needs of O&P users changed quickly.

*For complicated re-care evaluations, I wouldn’t do it [telehealth] just because there’s something that might be changing*. *(Leona)*

[In context of a child with plagiocephaly] *I would say it’s a little bit more important for the three-week check-in that they [the patient] are physically in the office because there are usually adjustments … to the helmet to accommodate for growth*… *(Ann-Marie)*

On very rare occasions, an initial subjective assessment via telehealth led to an inaccurate prescription. The subsequent in-person objective assessment highlighted that a different intervention would be more appropriate, which necessitated reapplication to an insurer.

*…there’s a patient who we did virtual care [video conference] for, they had an existing device*…. *They were recommended… a new set of the same device. But then when we saw them face to face [in-person], we’re like, "Oh wait, we need to do something else."..I think their spasticity went up.., so we had to go back and… resend for prior authorization. (Leona)*

#### Facilitators of an outstanding telehealth experience

Our interviews highlighted a myriad of things that practitioners did that add value and helped create an outstanding telehealth experience.

*Focus on the benefits of telehealth*, *not the limitations*. When practitioners focused on the benefits of telehealth, this helped others to see beyond its limitations.

Practitioners described several benefits of telehealth for O&P users including: access to a timely, convenient, and efficient O&P service that helped balance the competing demands of work, family, and healthcare. Given that telehealth helped improve access, practitioners noted there were fewer no-shows or last-minute cancellations.

*… if they [O&P user and their parents] can just stay home and take a phone call or email me a couple of pictures, that’s so much more time efficient* …*and family friendly, than demanding that they come to my office so I can tell them everything is okay. (Thomas)*

*I have a much lower no-show rate for those video visits because you’re calling their cell phone so they generally will pick up*. *(Ann-Marie)*

Practitioners felt that telehealth improved direct access for O&P users that, in turn, enhanced communication and patient-focused care. Telehealth was seen as less formal and made it easier to respond to simple queries without a real-time conversation.

*… what makes it [telehealth] successful is, it gives the patients the ability to ask questions on things that they might perceive as small, but that still are worrisome to them. …and that answer may be reassuring*…. *(Paige)*

*… I routinely provide that [follow-up via email or telephone] for patients because it’s often easier for everybody involved if we can solve something quickly via telephone or email*… *(Georgina)*

Many practitioners commented on other benefits to O&P users, such as reduced COVID-exposure for people with multiple comorbid health conditions.

*A lot of our patients are immunocompromised or just fragile, and they didn’t want to expose themselves by going out in the world, but we were able to touch base with them by doing telehealth*. *(Rebecca)*

For practitioners, the most common benefit was the efficiency that telehealth afforded. For example, telehealth made it easier to coordinate an insurance authorisation with the referring physician, prosthetist, and O&P user on the same video conference. Similarly, an ad-hoc review via telehealth helped practitioners prepare for a follow up in-person appointment by having the replacement components and a technician available.

*I [practitioner] have to get a doctor to sign off on everything I do. So having that access [via telehealth] to an actual doctor … it’s huge. It’s huge from a business perspective, as well as patient care too. …, it comes down to that timeliness … as I’m able to take care of the patient better and faster*. *(James)*

…. *something’s broken*, *and I’ll say*, *"Well*, *I have to order that before you come in*, *so I’m glad you showed that to me*.*" So*, *it definitely can save a visit by triaging it with a video [conference]*. *(Rebecca)*

Telehealth opened the door for multidisciplinary healthcare; particularly for practitioners working in the community where healthcare professionals were not geographically clustered. Telehealth also facilitated connection with a wide range of specialist practitioners (e.g., an upper extremity prosthetics specialist in another state) which helped enhance care.

*A lot of the rural areas don’t have those kind of physicians [physical medicine and rehabilitation doctors]… that can evaluate … a patient’s needs. So then we will have the patient in clinic with us and the doctor comes in [via] telehealth.* …*And then we all evaluate together… (Charlie)*

*[As a specialist practitioner]*, *if I can’t be there personally*, *the second-best thing is to do it by video*…*and we [treating prosthetist and specialist practitioner] can still treat the patient and make sure that they get what they need*. *(Reagan)*

Practitioners also described that telehealth gave them a better understanding of the O&P user’s home environment and family supports, as well as the ability to regularly connect with people who were housebound.

*That was really* … *beneficial, seeing my patient in their own environment. For example, one patient who uses a transfemoral … prosthesis, picking up a chair and moving it out of the way.… I’ve never seen her do that in the office because there was no reason [to], I would move it out of the way. (Lauren)*

*A patient focused attitude*. Practitioners overwhelmingly demonstrated a patient focused attitude. That willingness to preference the needs and interests of the O&P user, positively influenced the experience of telehealth.

…*we’re here to take care of our patients, and I think that offering them another option that, at $5 a gallon, saves them $200 in gas to come see me, it makes me aware that I’m doing everything I can to make decisions that are good for my patients. (Travis)*

*How much can you ask the parent to come*, *take off work and drive their four-year old five hours each way for a follow-up every two months*? *How much is that truly necessary*?… *that’s why the telemedicine helps in those areas*. *(Paige)*

Overwhelmingly, practitioners demonstrated their ability to meet O&P users where they were at, and the importance of giving people agency over the choice to use telehealth (or not). Similarly, practitioners shared examples that demonstrated that respect and dignity were key to a patient focused attitude and an outstanding telehealth experience.

*… there’s a lot of patients who come into the office who really wouldn’t have had to come, but they… like the personal contact and eyes on, or they’re uncertain because they’re new and they want the assurance*… . *that’s useful for the newer families*. *(Thomas)*

*We always ask them [patients]*, *“Do you want to do a video call*? *If so*, *what’s the best way*? *Teams*, *Zoom*, *FaceTime*, *or just a regular phone call*?” *We’ll ask them to lead that part*. *(Tess)*

A patient focused attitude was evident in how practitioners spoke of their desire to provide an outstanding telehealth experience, and go *above-and-beyond* to help ensure that O&P users were well supported.

*I’ve gone to patients’ homes when they have issues using telehealth and I’ve helped them set it up and run it to call the doctor*. *(Theo)*

*[In the context of a practitioner reassuring a parent] I say to them*… *“You are very competent and very capable*. *Now let’s work on this together”*. *That sort of cheerleading helps me to get them to do what needs to be done*. *(Maria)*

*High-level communication skills*. Our interviews highlighted that high-level communication skills helped provide an outstanding telehealth experience.

Some practitioners described the importance of building connection and a trusting working relationship—with O&P users, carers, or healthcare professionals—that facilitated candid conversations about the issues and built confidence that practitioners were genuinely there to help.

…*it’s not unusual for them [an allied health therapist] to just appear with the patient and the Mom. …. sometimes that is because … their [previous] orthotic was less than satisfactory, and so the therapist goes to be the overseer. I can tell when they come in, they’re very directive and very prescriptive, … and once they understand that I can perceive what matters for that child, you can just… watch their attitude melt and they relax. They become much more collegial. And we still talk about what they would like to see or what I think might be helpful, but the tension goes. (Thomas)*

While there was no one approach to building that trusting working relationship, practitioners spoke of the importance of being focused on the O&P user, present and attentive to the conversation, and deliberate in tailoring their communication to meet the needs of O&P users.

*I think it’s all about being able to communicate in a way that the patient can understand… Some patients are visual learners, some are auditory*. *So, I always try to do it [communicate] through words first, but if I get the sense that they don’t understand, then I’ll… go into different techniques. (Georgina)*

*[In the context of a video conference]*, *I try to focus on the patient and not other things going on around them*. *If I sit down and take notes*, *I make sure they can still see me [on the camera*] *(Eloise)*

Practitioners also spoke of the ability to effectively navigate difficult conversations. This required a willingness to truly listen to the issues without becoming emotionally invested. This seemed key to avoiding frustration, being able to ask thoughtful questions, and make considered decisions about the next steps.

*I think being able to articulate if there are issues, to not be frustrated about it, but like*, "*Oh you know this happens, but this is still why the telehealth is in your best interest”. (Leona)*

An outstanding telehealth experience was aided by practitioners who could clearly articulate what needed to be done during the consultation (e.g., how a camera should be positioned), and find alternative ways of expressing those needs when there was a communication breakdown.

*I’ve tried to coach people [patients] that* …*I would like you to use your cell phone [not your laptop], because if we’re going to use it like a camera, it’s much easier for you to manipulate the phone, to look at the foot… [and it] …is going to be the best quality. “…I want to be able to really see what’s going on.” (Rebecca)*

… *for those appointments [where a parent fits a device with the practitioner on video conferencing]*, *a practitioner … [would] have … a similar device on hand so we could hold it up to the camera and illustrate*, *"This is what we’re talking about*, *insert your screwdriver here to make this adjustment*.*” (Jackson)*

*Matching the people*, *purpose*, *and platform*. The ability of practitioners to choose which O&P users were appropriate for telehealth was key to an outstanding experience, as was the ability to match O&P users to the right telehealth mode given the purpose of the consultation.

Three factors seemed key to determining which O&P users were appropriate for telehealth: an established relationship between practitioner and O&P user, O&P users who were knowledgeable about their healthcare, and O&P users with the right supports available for an effective healthcare consult (as distinct from the technical support required to use telehealth). Each of these three factors will be discussed in detail in the following paragraphs.

An established relationship with O&P users gave practitioners a greater understanding of an O&P user’s needs, the likely issues, and the user’s ability to self-manage those issues (e.g., changing sock ply) or request help. There was no clear length of time to establish this relationship with case examples varying from a single initial consultation to a long-standing relationship built over many years.

*… I’m thinking more of our prosthetic patients that need more follow up. I’ll know that this person is fine, they’ll call me when they need something. Other people* … *maybe … more susceptible to having issues. (Owen)*

Practitioners noted that O&P users who were knowledgeable about their healthcare and had a lengthy lived experience with their orthosis/prosthesis, were able to accurately assess issues (e.g., which red marks were abnormal) and ask for help when they need it.

*I certainly offer it [telehealth] to the families who are on their second, third, fourth, fifth set of orthoses. They know exactly what they’re doing. They know what the red marks look like. They know how things should fit, and look, and feel and work*. *(Thomas)*

Practitioners noted that when O&P users were unable to independently manage their healthcare, the support of a parent, family member, carer, or healthcare practitioner (e.g., in a nursing facility) extended beyond simply setting up the telehealth consultation, and was critical given the O&P practitioner was not in-person.

… *I… have the family be really active and mark things up, and then put the brace back on right away…, so that I can give them possible tips of how to relieve that discomfort until they can get it adjusted by [an] orthotist in person. (Paige)*

While practitioners defaulted to using video conferencing or phone calls for scheduled consultations (e.g., initial assessment), they were deliberate in choosing the telehealth platform when problems needed escalation.

*Typically,* … *the patient would contact us. My office staff would… try to find out [what the problem was], or I would give them [the patient] a phone call… and then I would determine whether it was something that we could attempt to do FaceTime, or if they truly needed to come in …. (Molly)*

The ability to match the person, purpose, and mode required thoughtful risk assessment and clinical decision making on behalf of the practitioner. Our interviews engendered confidence that practitioners and O&P users could jointly make appropriate decisions about using telehealth; they weighed competing influences (e.g., the impost of travel, risk of an adverse event) to ensure the purpose of the consult was achieved, and that safe and effective O&P services were provided.

*So if they [the patient] live five hours away and they have an active therapist they’re working with who understands AFOs, I may put them on telephone follow-up*… *(Thomas)*

…, *the complexity of the device*, *the patient’s history with the device*. *If we’re just replacing something that they’ve had before*, *…we’ll discuss with the parents* … *whether or not they felt comfortable with having something sent to them and then fitting it with some telehealth guidance*… *(Jackson)*

*Setting up for success*. To provide an outstanding telehealth experience it was important that telehealth consultations were set up for success, starting with the systems and procedures of the clinical service.

Some practitioners and clinical services had dedicated considerable thought to the experience from the perspective of the O&P users, and how the well-developed systems that support in-person consultations could be replicated in a way that was appropriate for telehealth. There were several best practice examples including:

○ scheduling telehealth appointments rather than cold calling
○ blocking sequential appointments of a similar mode together to help practitioners transition from one appointment to the next
○ having an assistant set up video consultations in advance and resolve any technical issues
○ providing O&P users with clear and accessible information about the purpose of the consultation, what will happen during the consultation, and any preparatory instructions (e.g., please use a smart phone to make it easier to show your feet)

*[I tell my patients], they’re on my calendar as 30 minute appointments*… *that’s your 30 minutes. We’re going to try to call during those 30 minutes, so set the appointment at a time you can receive a phone call. (Thomas)*

*I ask that the staff gets that person [the patient] on 15 minutes beforehand just to make sure that we’re ready to go at that set time*. *(Alex)*

Practitioners recognised the need for administrative systems tailored to the unique risks associated with telehealth, such as an O&P user being left waiting on a video conference when a practitioner was delayed. Similarly, practitioners highlighted the need for processes that helped ensure that emails, texts, or phone calls got responded to in a timely way.

*… when a patient shows up [in-person], somebody says, "*… *your patient is in room three." When the patient’s waiting at home [for telehealth], I might be in room three with a different patient here, running over [time] and unaware of what time it is… (Rebecca)*

*… for our administrative staff*, *it’s challenging to know if we [practitioners] have done that phone call or not*, *because* … *we might not have documented*.. *it yet*. *(Tess)*

Given the different modes of telehealth in common use, it has become more challenging for practitioners to ensure that the medical record was accurate and up-to-date. Practitioners identified several best-practice examples, such as systems that made it possible to forward emails directly to medical records, or upload images to a cloud from a mobile device. Practitioners found it easier to reliably update a medical record when telehealth appointments were scheduled rather than ad-hoc calls, text messages and photos sent to a cell phone.

…*if it gets emailed directly in, the office staff will upload it into the patient’s record immediately. If we get a text, then we have to email the photo from our phone to the office for it to get uploaded, because we can’t upload it into the system from our phone. (Rebecca)*

Without the deliberate integration of telehealth into the existing systems or procedures of a clinical service, there were inevitably gaps in care.

*Sometimes with email, things would drop through the cracks. If you saw it, but didn’t respond to it right away, [you might then] forget to respond to it*… *(Eloise)*

*I think it’s hard whenever I’ve set aside that time [for a telehealth appointment] and I try to call the patient and*… *they don’t answer*. *I have to keep going because my schedule keeps going*. *Then when they call back*,… *I’m with someone else…*. *(Tess)*

#### Future proofing telehealth to make it sustainable

Many practitioners had a limited understanding of what telehealth was, which limited their ability to see its value, or engage with complex issues such as reimbursement or compliance with privacy regulation. While these issues didn’t seem to impact the quality of the telehealth experience, they will likely impact the sustainability of a well-regulated and high-quality telehealth service into the future.

*Practitioners’ knowledge about telehealth and its perceived value*. About one-third of practitioners perceived they did not use telehealth to provide O&P care, or viewed telehealth as synonymous with video conferencing. Many practitioners did not appreciate the extent to which they already used telehealth in the form of phone calls, emails, text messaging, or photo sharing.

… *communication with different patients through email is not really providing telehealth. (Theo)*

*When I look at telehealth*, *I look at video*, *that’s my take on it*. *(Stephen)*

Practitioners reflected that when leaders perceived little value in telehealth, it negatively impacted their availability to provide services using telehealth.

*I think the problem within my department, in leadership in particular, is that they don’t see the value in telehealth currently. So then, they are prohibiting or not helping to advance us to use that*. *(Lauren)*

*Reimbursement*. One of the most frequent issues raised by practitioners was reimbursement for telehealth services. Many practitioners described that they don’t get paid for telehealth consults given that reimbursement was based on delivery of the orthosis/prosthesis, not the clinical service provided. Many practitioners felt there was no incentive to provide services using telehealth without being able to invoice for the professional service.

*We get paid for the device, not our care*… *so the only billable appointment is a delivery appointment. So all of our evaluation and follow up is non-billable… (Johan)*

*Something has to be broken in order for me to bill time*.… *I can’t say*, *"And I spent 30 minutes educating them*.*" That doesn’t count*. *(Tess)*

By contrast, some practitioners acknowledged that the cost of the orthosis/prosthesis included the clinical service that went into the assessment, fitting, education and follow up appointments; irrespective of the mode used. Many of these practitioners acknowledged that one of the incentives for using telehealth was the efficiency that it afforded for many types of service encounters that were not able to be independently billed (e.g., an initial consult with a prospective O&P user).

*A lot of people treat it as a loss leader… but a necessary service*… *[In an in-person consult, you have] …that administrative time for the clinician and the front office staff dealing with a patient. That may be a 45–60 minute appointment by the time you’re done. Where if you can do it telephonically, you’re looking at 20 to 30 minutes, and you don’t tie up physical resources in a space constrained clinic. (David)*

*Regulation*. The second most frequently described issue for the sustainability of telehealth was the complexity of the current regulation given the requirements of federal, state, and profession-specific organisations. Practitioners noted that current policies require either an existing in-person relationship between a practitioner and O&P user before a telehealth consult, or that telehealth consultations are followed-up with in-person appointments.

*I think there’s a lot of restrictions in terms of ABC [American Board for Certification of Orthotics, Prosthetics and Pedorthics] and what we’re required to do, and I think that forces a lot of in-person*. *(Rebecca)*

*…you have to have a relationship with that patient before or during in order to have a virtual appointment*. *You can’t just meet the person for the first time virtually*.. *(Reagan)*

Many practitioners were worried whether the diverse telehealth modes currently in use met regulatory requirements, and the complexity of managing compliance given the lack of clear guidance and support in a rapidly changing landscape. For example, while a service provider might have mechanisms in place to ensure compliance with *the Health Insurance Portability and Accountability Act* (HIPPA)—such as outgoing email is encrypted—those mechanisms do not ensure the encryption of emails or text messages sent by O&P users to a service provider, nor do they extend to conversations by video conference using accessible, but insecure platforms.

*We do not have encrypted email to the office, and of course our phone use is not encrypted, and so we tell parents, "…I’m happy to receive your texts, but it’s going to my phone, it is not*… *encrypted…" (Rebecca)*

*I think we’re all learning because this availability [of telehealth] has come up so fast so I think there’s a lot that we don’t know*.… *certain platforms of video conferencing might not meet regulatory requirements in terms of privacy*….*(Georgina)*

A few practitioners spoke of the financial investment needed to ensure regulatory compliance for the sharing and management of protected health information including the provision of training, policy development, and establishment of technological safeguards to protect health information.

*… . the patients love that [i.e., communicating via text message]. I love that. But to do it compliantly it has to go into a system and that costs money*… *So, is it valuable enough to streamline things for your office to make it worth the cost? (Georgina)*

…*there are a number of platforms that are available now [for video conferencing]*, *but being able to do that in a way that … also integrate[s]*.. *into our EMR [Electronic Medical Records] system*. *… I can’t just have a video evaluation and attach it to the chart*. *… there has to be a technical way*.. *to do it effectively*. *[There is a]… cost of setting that system up because it doesn’t exist for us*. *(Charlie)*

## Discussion

Given the aim of this research, the following discussion has been sectioned to characterise the spectrum of clinical care provided using telehealth, and summarise the barriers and facilitators from the perspective of O&P practitioners. That discussion provides context for the key take-home messages including: creating an outstanding telehealth experience, making decisions about using telehealth, a right touch approach to policy and regulation, as well as the need for telehealth education and training. Lastly, we discuss our recommendations and avenues for further work.

### The full spectrum of O&P care is being provided using telehealth

In this study, practitioners shared examples of their clinical service which demonstrated that the full spectrum of O&P care was being provided using telehealth including: initial consults, education, delivery of an orthosis/prosthesis, and scheduled or ad-hoc review appointments; as illustrative examples. While these examples may contrast with the perception that O&P is a *hands-on* profession not well suited to telehealth, these observations extend previous research describing the diverse ways that telehealth is used to provide O&P services [10]. While we are not aware of other O&P studies that report on the variety of clinical services provided using telehealth, there are examples in the broader allied health literature where telehealth has been used in similarly *hands-on* disciplines such as physiotherapy [42, 43] as well as in different settings [44, 45].

### Barriers and facilitators

The results of this study highlight that, from the perspective of the practitioner, the barriers and facilitators to a high-quality telehealth service can be considered along a spectrum from basic needs to value adds. For example, practitioners described some absolute essentials needed to participate in telehealth such as access to reliable phone or internet access and contemporary communication technologies. Once the absolute essentials were in place, both practitioners and O&P users needed the capability to use them independently or access to supports (e.g., to solve the inevitable technical challenges). There were several facilitators that transformed a satisfactory telehealth experience into an outstanding one including: practitioners with high-level communication skills, and practitioners that could thoughtfully match the purpose of the consult to the appropriate telehealth mode given the needs of the O&P user.

Many of the barriers and facilitators summarised by these themes are reflected in the broader body of telehealth literature. For example, many studies highlight similar barriers to participating in telehealth given the technological challenges faced by many older people, those with cognitive decline, vision or hearing impairments [13, 23, 46–48]. Many studies also highlighted similar facilitators such as focusing on the benefits of telehealth, rather than the limitations, citing benefits including seeing patients in own home [23, 47] and the ability to co-ordinate virtual consultations with multiple practitioners [13, 23, 47, 49]. While practitioners appreciated the many benefits of telehealth, they also recognised that telehealth needed to be thoughtfully chosen to meet the needs of the patients and the purpose of the consultation [23, 47, 50].

### An outstanding telehealth experience

Consistent with previous research [50, 51] and published guidelines [52, 53] our interviews highlighted examples of best practice that helped to create an outstanding telehealth experience. For example, some clinical facilities had considered the experience of O&P users, and the systems needed to ensure a high-quality telehealth experience including: scheduling telehealth appointments, providing instruction and support to O&P users, and establishing administration process that facilitated timely responses and updating of the medical record.

Looking beyond what clinical facilities can do, many practitioners created an outstanding telehealth experience by focusing on the benefits that telehealth could provide rather than its limitations [14, 51]. These practitioners considered when telehealth was appropriate to meet the purpose of the consultation and offered O&P users agency over the decision to use telehealth, [10, 14, 50]. By adopting a genuine patient-focused attitude, these practitioners provided many of the benefits of telehealth to O&P users in their care such as convenience, reduced travel, or the ability to balance the competing demands of work, family, and healthcare [10, 11, 14].

While these aspects of best practice were reported from the practitioner perspective, they mirror the perspectives of O&P users [10] and other patient cohorts [11, 14, 48] which suggests these experiences with telehealth are likely universal. For example, technical issues that affected the reliability of telehealth for practitioners [23] also detracted from the experience of O&P users [10] and patients across a range of settings [12, 14, 46]. Similarly, having agency and control over the choice to use telehealth was important for both practitioners and O&P users, as was the convenience that telehealth afforded [10, 11, 14, 48, 49].

### Practitioners and O&P users can make appropriate decisions about telehealth use

Given that telehealth was being used across the entire spectrum of O&P services, it was reassuring to observe practitioners consistently make considered decisions about the appropriateness of the telehealth mode given the purpose of the consultation [54]. Even in cases where the use of telehealth may be controversial (e.g., remote fitting of a device) practitioners had compelling reasons for the clinical decision (e.g., COVID-related lock downs of skilled nursing facilities) and weighed the risks (e.g., risk of poor fit and skin breakdown) against alternatives (e.g., delaying O&P care may increase the risk of a fall). Practitioners also consistently put in place risk mitigation strategies appropriate to the clinical scenario and the supports available (e.g., a nurse practitioner reviewing the fit of the orthosis/prosthesis). The capability of practitioners to make thoughtful decisions about the appropriateness of telehealth and put in place risk mitigation strategies, has been observed in other studies [10, 47, 50].

### Right-touch approach to policy and regulation of telehealth

Given that practitioners and O&P users consistently made considered decisions about when telehealth can be used, for what purpose, and for whom, we could find no evidence to support guidelines or policies that require telehealth consultations to be punctuated by in-person consultations, or similarly risk-averse approaches.

Consistent with previous research [55–58], we believe that a *right-touch* approach to policies that regulate practitioners is needed; one that empowers practitioners to make considered decisions in accord with their scope of practice, where the relative risks of different modes of service are considered. By considering telehealth as just another mode of service, we recognise that all modes of O&P service carry risks, even if we do not perceive them initially.

In countries with well-developed practitioner regulation, practitioners have the capability to work autonomously and make decisions about the service they provide in keeping with their professional and personal scope-of-practice [53, 57, 59]. In countries with less well-developed practitioner regulation, where the scope of clinical practice maybe more limited, practitioners may need support and training to move toward more autonomous clinical decision making that includes decisions about the use of telehealth [60].

While we encourage organisations responsible for regulating practitioners to adopt evidence informed policies that do not restrict the use of telehealth, this will have limited impact until those policies align with funding agencies as well as federal- or state- healthcare regulators [58, 61]. As participants in this study noted, telehealth regulation in the USA is complex [62] which negatively impacts the way O&P services are provided.

### Telehealth-specific education and training

The need for education and training that can support a high-quality telehealth experience is evident in our observation that a proportion of practitioners did not have an accurate understanding of telehealth [47]. These practitioners were often the least likely to offer telehealth to their patients, and often had narrow views about how telehealth could be used effectively [51]. When these practitioners were in leadership roles, they were often the least engaged in addressing some of the more challenging problems relating to legislative compliance, privacy, and the policies and procedures needed to support a high-quality telehealth experience.

### Recommendations

There are several recommendations that arise from this work.

Practitioners and clinical services should consider adopting many of the best-practice examples reported in this research including: scheduling telehealth appointments, providing O&P users with clear instructions so they can set themselves up for the specific appointment, embedding processes that ensure timely communication and developing policies that govern how health information and images are received and stored.

Access to telehealth specific education and training will be key to the provision of a high-quality O&P service using telehealth in the years ahead [23, 51]. There are opportunities for professional associations and regulators to encourage participation in telehealth education by offering continuing professional development activities or credits for completing telehealth training provided by a third-party such as telehealth providers [63] or educational institutions [64, 65].

Given the need for a right-touch approach to policy and regulation, there are opportunities to simplify the complex regulatory environment and thereby reduce barriers to the use of telehealth [46, 61, 62]. Simplifying regulation will require policy makers and regulators at all levels to adopt evidence-informed positions that reflect the true risk—not the perceived or potential risk—of providing safe and effective O&P services using telehealth. In well-regulated environments, we encourage reliance on established mechanisms—scope of practice, code of conduct, complaints mechanism–to provide the guardrails for telehealth, just as these mechanisms support safe and effective services in other modes [62, 66].

The profession requires a clear and strategic position on reimbursement for telehealth. While reimbursement was consistently described as a barrier to the provision of telehealth [46, 61], practitioners have been providing O&P care using telehealth for many years within the current reimbursement mechanisms. As such, there is little impetus for reimbursement agencies to change the status quo without a compelling reason to do so. Unfortunately, the current debate about reimbursement seems focused on whether practitioners should be reimbursed for telehealth as a discrete activity, or as part of the overall cost of the O&P care. In our view, there is a more strategic conversation about reimbursement where the goal should be to support high-quality and well-regulated telehealth services that helps more people access the safe and effective O&P care they need. Our goal should be to advocate for the financial investment that allows clinical facilities to establish policies and procedures that support high-quality telehealth, as well as technology and software solutions to solve challenges such as privacy and simpler medical record keeping.

### Future work

Given the opportunity to translate the findings of this work, we have published a telehealth guide specific for the O&P profession [S2 File]. The 23-page guide provides high-level information that: defines telehealth, characterises how telehealth is being used across the spectrum of O&P services, describes what clinical service providers and practitioners can do to set up consultations for success, and the challenges to achieving high-quality and well-regulated telehealth services into the future. We hope that regulators, professional associations, educational institutions, and clinical facility owners/managers will use the guide to promote greater awareness of telehealth and, over time, improve access to high-quality telehealth services.

In terms of future research, we see opportunities to better understand the experience of practitioners outside the USA and to use that knowledge to tailor recommendations for different practice settings. This approach may help reconcile the different experience of using telehealth and build a more nuanced conversation about what is required to provide safe and effective O&P services aligned to the requirements of different practice settings; acknowledging the variation in regulation, funding, and scope-of-practice for telehealth globally.

Given the limited literature specific to the use of telehealth in O&P care, there are opportunities to further research in this area, including studies designed to compare efficacy, cost effectiveness, and patient outcomes between telehealth and in-person modes of O&P care. Fortunately, syntheses of literature in similar populations can help inform the specific hypotheses and study designs [14].

### Limitations

Given this research was conducted in the USA, we acknowledge that the perspectives and experience of the practitioners we interviewed may not reflect the experience of those outside the USA. As such, care should be taken when generalising these findings of this research to other practice settings.

We recognise that those who responded to our invitation to participate are likely those who already use or have an interest in telehealth. The experiences and views reported in this research may not be reflective of the wider profession that includes people with little to no experience using telehealth. That said, it was important that we interviewed practitioners who could speak authentically about the barriers and facilitators to using telehealth.

We have been deliberate in omitting participant-level details and using pseudonyms to help protect the privacy of participants and the organisations they work for.

## Supporting information

S1 Checklist COREQ (COnsolidated criteria for REporting Qualitative research) checklist. (PDF)

S1 File Semi-structured interview guide. (DOCX)

S2 File Telehealth for the orthotic and prosthetic profession. A 23-page, full-colour guide to promote awareness of high-quality telehealth for practitioners, clinical facilities, regulators, professional associations and educational institutions.
(PDF)

S3 File (DOCX)
