# Supplementary material for: Exploration of the barriers and facilitators influencing use of telehealth for orthotic/prosthetic services in the United States of America: An orthotist/prosthetists perspective
Source: PLoS One. 2024 Oct 24;19(10):e0309194. doi: 10.1371/journal.pone.0309194 (PMC11500877; doi:10.1371/journal.pone.0309194)
Supplement: S1 File — (DOCX) [file pone.0309194.s002.docx]

**Semi-structured Interview Guide**

***Project title:* Exploration of the barriers and facilitators influencing use of telehealth for orthotic/prosthetic services in the United States of America: an orthotist/prosthetists perspective.**

**INTRODUCTION**

Hello [*insert participant name],*

Great to meet you, I’m *[insert researcher name].*

Thank you for making the time to talk me today. As you’re aware, I am part of a research team who are investigating the use of telehealth orthotic/prosthetic services, in the United States. My team is made up of people from La Trobe University, and the University of South Australia.

During our interview, I would like to learn more about who you are, so I will be asking you a series of short demographic questions, such as [clinical experience, age, type of practice]. We will then spend most of our time exploring in more detail, your thoughts about the use of telehealth to provide O&P services

We learn the most from interviews that are more like a conversation, so please feel free to be open in describing your perspectives. The interview will be audio recorded so that we can transcribe and analyse the data. Your responses will be combined with those from lots of other people that we are interviewing, and we will be looking for common experiences.

I expect the interview will take about 30-45 minutes.

Do you have any questions for me at this stage?

**PRESS RECORD**

Ok, I am going to go ahead and start recording our interview.

**HOUSEKEEPING**

Before we begin, I have a few house-keeping matters to work through:

- For our recording, would you please **state your name**?
- I need to make sure that you’re **eligible** to participate, so could you confirm for me that you are a certified orthotist and/or prosthetist in the United States of America? [y/n]

Thank you.

- I also need to make sure you **understand the purpose of the study** and what will happen in this interview. Would you please briefly describe your understanding of the study and today’s interview? [ensure paraphrase is accurate]

Thank you.

- Finally, do you **consent** to take part in this interview? [y/n]

Thank you. Ok, let’s begin.

| **Main questions** |  |  |
| --- | --- | --- |
| **Topic One: Demographics** | | |
| To start us of, I have a series of demographic questions to ask you. The purpose of these questions is to help us understand who the participants are, who are taking part in this research which will help us to interpret our findings. | | |
| What is your age? |  | |
| What is your gender? (M/F/other) |  | |
| In which state do you currently live or work? |  | |
| Zip-code |  | |
| **Topic Two: Clinical and telehealth experience** | | |
| I’d like to understand more about your day-to-day practice as an orthotist/prosthetist: | | |
| Do you currently practice orthotics and prosthetics *clinically*, as part of your role?  Can you estimate % time in different roles (such as clinical orthotics/clinical prosthetics/ management/admin/research/sales) |  | |
| How many years have you been practicing in this discipline? |  | |
| Are you to happy to provide the name of the organisation that you work for? We would use this information to ensure our participants are as diverse as possible. |  | |
| Type of organisation (public hospital, private practice, other) |  | |
| Orthotic/prosthetic/both?  Adults/Paeds/both?  Any specialities? |  | |
| Size of organisation (estimated FTE clinical staff, technical staff, admin staff) |  | |
| Part of conglomerate or independent? |  | |

| **Main questions** | **Additional questions** | **Clarifying questions** |
| --- | --- | --- |
| **Topic Three: Barriers to telehealth** | | |
| Thankyou, that concludes all the demographic questions that we’d like to ask.  Next, I’d like to talk about your perspectives on the barriers to using telehealth to deliver orthotic/prosthetic services. When we talk about ‘barriers’ what we mean are those things that make it more difficult to use telehealth, or stop you from using telehealth altogether. | | |
| Have you ever used telehealth to provide orthotic/prosthetic services?  *Telehealth is defined as using any non-face-to-face method to provide clinical services, so this could include: email, telephone calls and courier services.* | *Try to organise these into different activities, and address each one separately. Record them here to make sure you don’t forget, and it will help with the summary:*  *Ask about frequency number of times per month:*  *Perhaps dig into age – is it really harder to use telehealth for older people? How?* | |
| You’ve noted that you *have* used telehealth to provide orthotic/prosthetic services  or  You’ve noted that you *have never* used telehealth to provide orthotic/prosthetic services. | Can you tell me more about your **previous experiences** using telehealth to provide orthotic/prosthetic services?  What was the **purpose** of the appt?  What **sorts of services** have you delivered?  What **mode of telehealth** did you use?  **Who** did you deliver these services to? (describe the consumers)  What **worked and why?**  What **didn’t work and why**?  When do you think it is **appropriate** to use telehealth, and when do you think it is **not appropriate?** Why?  How **satisfied** were you?  Do you think there were any **barriers** to you using telehealth?  Do you have any thoughts on **why** you have never used telehealth?  Can you identify any **barriers**?  *PROMPT: Barriers could be at the practitioner level, consumer level, organisational level, state/funding body level?*  *Barriers could be systemic (system level) or cultural, or context specific.* | **PROMPTS**   - Can you expand on that – tell me more? - Can you tell me why? - Why do you think? - Can you give me an example? |
| **Topic Four: Facilitators of telehealth** | | |
| Ok, so we have talked in depth now about the barriers – so these are things that make it difficult or impossible to use telehealth to deliver orthotic and prosthetic services.  Now I’d like to shift our thinking to look at the opposite – the facilitators. So these are things that make it easier to use telehealth. | | |
| You’ve noted that you *have* used telehealth to provide orthotic/prosthetic services  OR  You’ve noted that you *have never* used telehealth to provide orthotic/prosthetic services. | We talked about the barriers – the things that made it more difficult to use telehealth previously. Can you think about what, if anything, **could have made it easier** to use telehealth?  What **needed to change** about those circumstances to reduce the barriers?  We talked about the barriers – the things that stopped you using telehealth previously. Can you think about what, if anything, **could have helped** you to be able to use telehealth?  What **needed to change** about those circumstances to make it possible? |  |
| Thinking about the **future**, do you think you are likely to use telehealth to provide orthotic/prosthetic services? | If **yes – why**?  If **no – why**? What’s stopping you? |  |
| **Open ended** | | |
| Is there anything else you **would like to add** that we might have missed, or that you think is important? |  | |

**SUMMARISE KEY POINTS**

Before we wrap up, I would like to just take a minute to summarise what I hear are the key points from our discussion today:

[summarise key points]

Does this sound like a fair summary of our discussion?

**CONCLUDE INTERVIEW**

Thank you for your time today. We appreciate that while there is no direct benefit to you in taking part, your perspective does contribute to the development of a best-practice guide for telehealth services in orthotics and prosthetics.

The next steps are that we will be analysing each participant’s interview and draw out common themes. We will send you a copy of the themes and quotes from your interview to ensure we’ve understood the detail of your perspective. You will be able to provide feedback if required and then we can make adaptations.

Thank you again for your time, and have a lovely rest of the day.

**END INTERVIEW AND RECORDING**
