# Supplementary material for: Exploration of the barriers and facilitators influencing use of telehealth for orthotic/prosthetic services in the United States of America: An orthotist/prosthetists perspective
Source: PLoS One. 2024 Oct 24;19(10):e0309194. doi: 10.1371/journal.pone.0309194 (PMC11500877; doi:10.1371/journal.pone.0309194)
Supplement: S2 File — A 23-page, full-colour guide to promote awareness of high-quality telehealth for practitioners, clinical facilities, regulators, professional associations and educational institutions. (PDF) [file pone.0309194.s003.pdf]

TELEHEALTH

FOR THE ORTHOTIC & PROSTHETIC PROFESSION

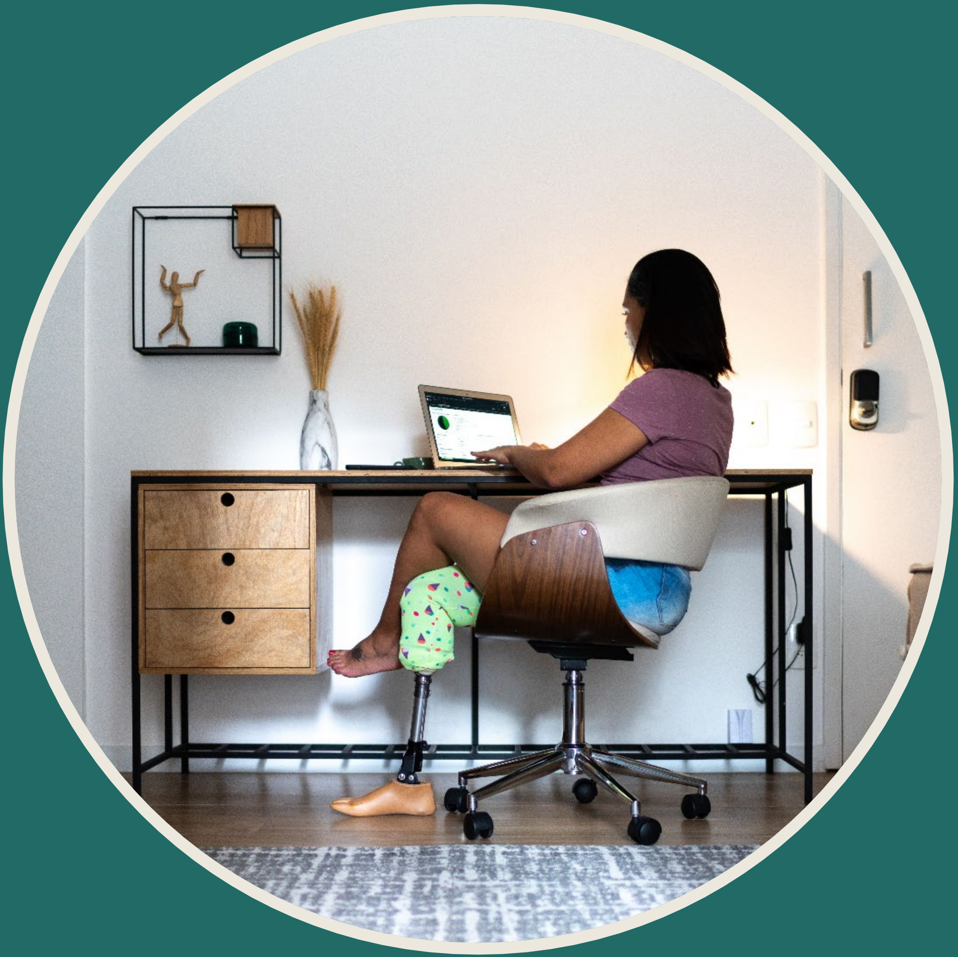

Jessica Landers, BPO
Leigh Clarke, MPH
Saravana Kumar, PhD

Emily Ridgewell, PhD
Katie Bishop, B. Psyc
Michael Dillon, PhD

About this guide

This telehealth guide, and the peer reviewed research upon which it was based, were funded by two grants from the American Orthotic Prosthetic Association and the Centre for Orthotic and Prosthetic Learning (EBP-043020, EBP-043021).

The evidence-base for this guide comes from our published research:

Dillon et al. (2023) Disabil. Rehab. doi.org/10.1080/09638288.2023.2196094
Dillon et al. (2024) PLoSOne. doi.org/10.1371/journal.pone.0309194

The illustrative first-person quotes in this guide may differ slightly from the original research. In editing quotes so they stand alone, every effort has been made to preserve the original meaning.

Cover image has been licensed from iStock, photo ID: 1306016265

We are grateful for the support of an Expert Advisory Group who supported the development of this guide. Their names have been used here with permission:

Cassandra Delgado, MSPO, CPO
Annie J Hill, PhD
Joseph McTernan
Stephen Fletcher, CPO
Michelle Hall, MS, CPO
Shane R Wurdeman, PhD, CP, FAAOP

If you have any queries about this telehealth guide, please contact:

Dr. Michael Dillon, PhD
Professor of Prosthetics and Orthotics
La Trobe University. Australia, 3086
Email: michael.dillon@latrobe.edu.au

This version of the guide was published on: 18th August 2024

Suggested citation:

Landers J, Ridgewell E, Clarke L, Bishop K, Kumar S, and Dillon MP (2024) Telehealth for the orthotic prosthetic profession. In: Dillon et al. (2024) PlosOne. (Suppl.) doi.org/10.1371/journal.pone.0309194

Disclaimer

This guide is for general informational purposes only. It is not intended as a substitute for medical or legal advice. Nor is this publication exhaustive of the subject matter. The information set out in this publication is current at the date of publication. Persons implementing any recommendations contained in this publication must exercise their own independent skill or judgement or seek appropriate professional advice relevant to their own particular circumstances. Accordingly, the authors have no liability to any users of the information contained in this publication for any loss or damage, cost or expense incurred or arising by reason of any person using or relying on the information contained in this publication.

Welcome!

We're absolutely delighted that you've picked up this telehealth guide specifically written for the orthotic and prosthetic (O&P) profession!

Over the last three years we've had the privilege of conducting two studies to better understand the barriers and facilitators to telehealth from the perspective of O&P users, as well as orthotists/prosthetists.

In developing this guide, we have sought to share the most important lessons from our research **to promote high-quality telehealth practice across the O&P profession.**

We hope that the guide serves as a valuable and accessible introduction to help you learn more about telehealth, develop your capability, and appreciate many of the complex issues we'll have to solve as a profession to ensure high-quality telehealth practice in the years ahead.

The guide is not intended to be a prescriptive set of rules or guidelines about who telehealth is suitable for, or what clinical services can be provided by telehealth. Based on our research, prescriptive approaches are not appropriate in well-regulated healthcare settings. Instead, we hope the guide promotes good clinical judgement where practitioners are empowered to consider telehealth given an understanding of the purpose of the consult; the preferences, capabilities, and supports available to the O&P user; as well as the risks involved with choosing either telehealth or an in-person mode.

We imagine this guide will be of value to orthotists and prosthetists, managers/owners of clinical facilities, educators, as well as associations responsible for regulating the profession and setting professional practice standards.

I'd like to acknowledge and thank all those who participated in our research and shared their experience of telehealth. This work wouldn't have been possible without the research grants made available by the American Orthotic Prosthetic Association and the Centre for Orthotic Prosthetic Learning. I'd like to thank members of the expert advisory group who helped to steer the development of this guide. Lastly, I'd like to thank the members of the research team who've contributed their time, expertise, and talents to both the peer reviewed research, and this guide specific for the O&P profession.

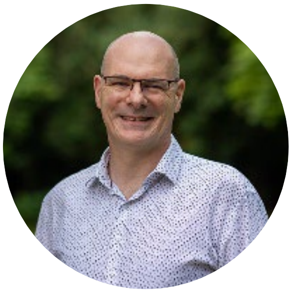

Professor Michael Dillon, PhD.
La Trobe University

Contents

Topic	Page
Welcome	3
Contents	4
What is telehealth?	5-6
How is telehealth used in O&P?	7-8
Why choose telehealth?	9-10
Professional standards	11
Setting up for success – clinical facilities	12-15
Setting up for success - practitioners	16-18
When is telehealth suitable?	19-20
Future-proofing telehealth	21
More information	22

What is telehealth?

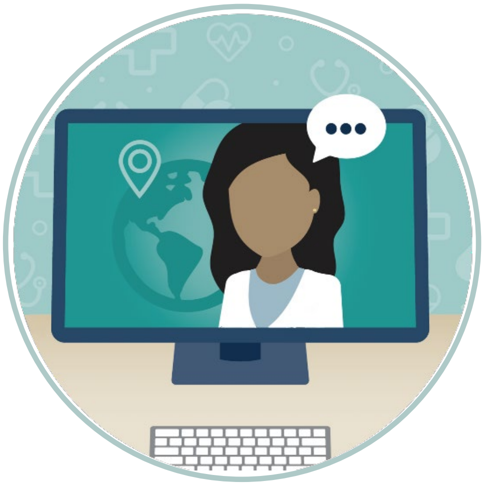

"Telehealth: The delivery of healthcare where a patient and healthcare professional are remote from one another."

- New England Journal of Medicine, Catalyst. (2018)

While there are many ways to define telehealth, technology now plays an important role in connecting patients and healthcare professionals for the purpose of providing high-quality healthcare.

Some people don't think that high-quality O&P care can be provided by telehealth. Our research shows that telehealth is being used to provide safe and effective O&P services across the whole spectrum of care, from initial consultations to regular reviews.

What is telehealth?

A range of **modes** were used to provide telehealth services across the O&P profession.

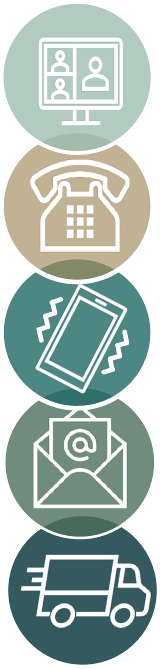

VIDEO-CONFERENCING

LANDLINE CALLS

CELL PHONE CALLS & TEXTS

EMAIL

COURIER SERVICES

Many orthotists and prosthetists do not recognise all these modes as ‘telehealth’, instead they considered telehealth to be synonymous with video-conferencing. In contrast, our research shows that all these modes are being used across the full spectrum of O&P care, from initial consultations to regular reviews.

Through our research we saw many examples of high-quality O&P care where orthotists and prosthetists carefully evaluated-risk and made thoughtful decisions about the appropriateness of telehealth.

Telehealth in O&P

Orthotists and prosthetists told us that **video-conferencing** was the most common telehealth mode used to provide O&P services, followed by **phone calls**, **texts**, **email**, and **courier services**.

INITIAL CONSULTS

Initial consults, such as meeting a potential patient, typically occurred over video-conferencing or phone.

INTERDISCIPLINARY CARE

Orthotists and prosthetists, referrers, prescribers and specialists, used video-conferencing to connect with each other and with a patient, to ensure everyone was on the same page.

SUBJECTIVE ASSESSMENT

An initial subjective assessment to understand a patient's condition, goals and type of intervention, often took place using video-conferencing, and before an in-person consult.

Telehealth in O&P

DELIVERY

In rare cases, when in-person consults were not possible, remote delivery/fitting of a device took place, with an orthotist or prosthetist and patient connecting over video-conference.

TROUBLE-SHOOTING

When problems arose, patients connected with orthotists and prosthetists by phone, texts, or emails. Often video-conferencing was used to further investigate the issue.

ROUTINE FOLLOW-UP

Orthotists and prosthetists used phone or video-conferencing to connect with patients for routine follow-ups.

COURIER SERVICES

Consumables, such as replacement prosthetic liners or socks, were often couriered to patients. On rare occasions, devices were couriered to another health professional who undertook the delivery/fitting of a device with the support and oversight of an orthotist or prosthetist who joined over video-conference.

Why choose telehealth?

There are many **benefits** of **telehealth for O&P users**. Telehealth facilitated timely, convenient, and efficient services that helped balance the competing demands of work, family, and healthcare.

IMPROVED ACCESS

Telehealth facilitated access to O&P services and specialists.

EFFICIENCY

Telehealth improved the efficiency of O&P services and made follow-up in-person consults more efficient.

“..a lot of times we're having trouble getting the right kind of doctor … so then we will have the patient in clinic with us and the doctor comes in telehealth.“

“It’s just easy to send somebody some socks or some liners or some rubber bands...to get them going right then and there. “

Dillon et al. 2024

“We can call, we can know the need, we can have it here and have a very efficient visit for them when they come.”

“...they spent a total of between one hour and 11 hours to come tell me we didn't need to be here.“

“...if I can get insurance information, then we can at least get started on verification earlier, before I even see them.”

Why choose telehealth?

CONVENIENCE

Patients were able to choose a service mode that helped them balance life's demands.

"If they can just stay home and take a phone call or email me a couple of pictures, that's so much more time efficient, and safe, and energy efficient, and family friendly, than demanding that they come to my office so I can tell them everything is okay."

"We'll try for their convenience to do a video telehealth, and especially children with disability, their lives are complicated enough. They don't need to be doing unnecessary road trips if they're fine."

PATIENT-CENTERED CARE

Telehealth helped orthotists and prosthetists provide timely, efficient and patient-centered care.

"I think that offering them another option that saves them $200 in gas to come see me. It makes me aware that I'm doing everything I can to make decisions that are good for my patients."

"...what makes telehealth successful is, it gives the patients the ability to ask questions on things that they might perceive as small, but that still are worrisome to them."

Dillon et al. 2024

Professional standards

To ensure the delivery of safe and effective services, orthotists and prosthetists must adhere to the **standards and codes** set out by their certifying body. While the names for these standards and codes will vary by jurisdiction, we suggest that **the same standards required for an in-person consultation should apply to any telehealth consultation.**

Orthotists and prosthetists should be **aware of their obligations** including:

- **Professional responsibility**
- **Scope of practice**
- **Facility accreditation**
- **State licensure**
- **Privacy and cybersecurity**
- **Consent**
- **Reimbursement**

Setting up for success

CLINICAL FACILITIES

A **telehealth service model** describes the five key commitments that clinical facilities can make to help ensure O&P users have access to high-quality care, that is both safe and effective.

MISSION

Commit to doing telehealth well by embedding this commitment into your organization's mission, vision or charter. When leaders are committed to telehealth, they are better able to find solutions to some of the complex issues that prevent provision of an outstanding telehealth service.

POLICIES & PROCEDURES

Implement systems that integrate telehealth into the everyday norm such as policies that help ensure safe and effective practice, procedures that facilitate scheduling, and systems for good medical record keeping.

Setting up for success

CLINICAL FACILITIES

SERVICE CULTURE

A service culture must be explicitly patient-focused. This ensures O&P users have a great telehealth experience, where their needs are met in ways that are safe and clinically appropriate.

HUMAN RESOURCE CULTURE

A workplace culture must value telehealth as a tool to facilitate the provision of high-quality O&P care, and support staff to use telehealth and use it well. This support might look like access to education, training and resources.

PHYSICAL ENVIRONMENT

Staff must have access to appropriate information technology and a place conducive to high-quality telehealth appointments. This might include up-to-date hardware and software that is privacy-compliant, as well as a quiet and well-lit space for conducting video-conferencing and telephone calls.

Setting up for success

CLINICAL FACILITIES

We've seen examples of **outstanding telehealth service,** where clinical facilities have deliberately set-up their organization for success.

- Policies explicitly focussed on telehealth
- Minimising barriers that prevent the clinical facility from using telehealth, or from using it well
- Systems for scheduling telehealth appointments in the same way as in-person appointments
- The same standards for medical record keeping across all modes of care
- Staff who share a commitment to a high-performing telehealth culture
- Patients can access high-quality services in a way that is timely, convenient and best meets their individual needs
- Support for staff to deliver high-quality O&P services using telehealth (e.g., through specific training).

Dillon et al. 2024

Setting up for success

CLINICAL FACILITIES

Our research highlights examples of outstanding telehealth service, where **clinical facilities have set-up their telehealth consults for success.**

"You need to set up a dedicated space for video calls, or dedicated times that it's done, so that you can be efficient and do them one after the other, or have practitioners rotating through a single dedicated space."

"I ask that the staff gets that person on 15 minutes beforehand just to make sure that we're ready to go at that set time. They have the person get on early, make sure that they're there and we're ready to hit the ground running."

"We actually have a secure cloud, like an electronic medical record that you can request file uploads. We discourage families from sending photos via email. Especially in scoliosis, a lot of the photos that we need are essentially in bra and underwear, back exposed. We send them a secure link that they can upload those photos to allow us to view the patient in their brace".

"I would have dedicated times for telehealth, as opposed to let's schedule an appointment at any time, because it's very disruptive for me to go from Zoom meeting, to a clinic room, to a lab, to a hospital - being pulled 15 different directions. It allows me to organize my time more efficiently. I would stay with a single platform, instead of multiple different platforms. It just makes the process cleaner and simpler, for me as a busy clinician"

Dillon et al. 2024

Setting up for success

PRACTITIONERS

We've seen examples of **outstanding practice** where orthotists and prosthetists have **deliberately set-up their telehealth consults for success**, in much the same way as in-person consults.

- A patient-focused approach and commitment to building an authentic relationship
- Empowering patient choice and decision-making through educating patients about their health condition to facilitate a shared language
- Really understanding the patient, including: their motivation for telehealth, how it can benefit them, and whether they have access to technology and the capability to successfully use different telehealth modes given the supports available.
- Scheduling telehealth appointments instead of cold-calling
- Clearly communicating the purpose of the consultation to the patient in advance, and describing what will happen during the consultation
- Clearly communicating how the patient needs to set up for the telehealth consult (e.g., could we use a mobile phone rather than a desktop computer because I need to be able to see you stand and walk, or see the spot on your foot).

Dillon et al. 2024

Setting up for success

PRACTITIONERS

Our research highlights examples of outstanding telehealth practice, where **orthotists/prosthetists have set-up their telehealth consults for success.** A patient focussed approach, great communication and patient education were key.

"I try to focus on the patient and not other things going on around them. If I have to sit down and take notes, I make sure they can still see me [on the camera]."

"We always ask them [patients], "Do you want to do a video call? If so, what's the best way? Teams, Zoom, FaceTime, or just a regular phone call?" We'll ask them to lead that part."

"How much can you ask the parent to come, take off work and drive their four year old five hours each way for a follow up every two months? How much is that truly necessary? And so that's why the telemedicine helps in those areas."

"And so I actually have the family be really active and mark things up, and then put the brace back on right away and try to see if we can see on the inside of the brace where it marked, so that I can give them possible tips of how to relieve that discomfort until they can get it adjusted by orthotist in person."

"We ended up asking parents to do the assessment in the patient's bedroom, because their telehealth setup would usually be in an office with a desk and it was difficult to get enough space to see the patient - if the patient was ambulatory, to have them walk more than three feet."

Dillon et al. 2024

Setting up for success

PRACTITIONERS

Our research shows that practitioners and patients jointly made appropriate decisions about using telehealth, given the interplay between the needs of the **patient**, the **mode**, and the **purpose** of the consult.

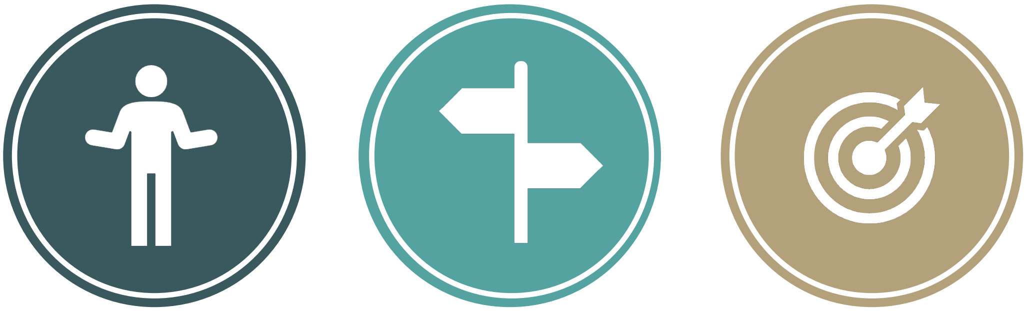

PATIENT + MODE + PURPOSE

- Patients who were knowledgeable about their own health care were better able to accurately represent and advocate for their own needs.
- Patients must have access to reliable reception, the required IT and software, as well as the capability or support to use them.
- Practitioners and patients need to determine whether the purpose of the consult can be safely and effectively achieved using a telehealth mode, or an in-person mode.

When is telehealth suitable?

The decision about when telehealth is (or isn't) appropriate requires **clinical judgement,** underpinned by an understanding of: the purpose of the consult; the patient's preferences, capabilities and supports, any payer or regulatory limitation; as well as the risks involved with choosing either telehealth, in-person, or no treatment.

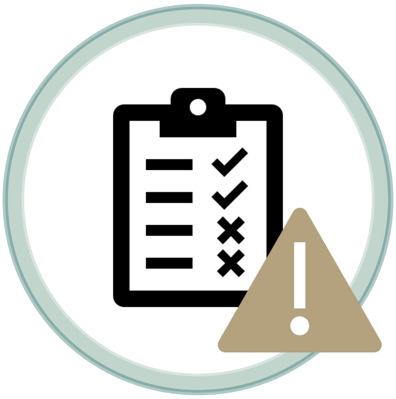

While we often default to thinking about the risks associated with telehealth, there are risks associated with in-person consultations too, as well as not providing treatment. Hence, why it is important the practitioner can effectively weigh the myriad of factors that must inform their decision, whilst ensuring the same standards required for an in-person consultation apply to any telehealth consultation.

When is telehealth suitable?

Our research shows that **practitioners have the capability** to weigh complex considerations, and make thoughtful **decisions about when telehealth is appropriate** (and when it isn't).

"So if the patient lives five hours away and they have an active therapist they're working with who understands AFOs, I may put them on telephone follow-up... "

"Patients who I may not offer telehealth to would be, for instance, amputees, who are unreliable about their use of the prosthesis or who have some cognitive impairment and really need that face-to-face visit and assessment. Patients where I really want to see the skin, and patients where I know they can't go one visit without needing adjustments."

"If you have a good understanding of a patient and a family, and that's one of the nice things about peds is we tend to see these patients an awful lot for an awful long time, and you get a pretty good idea of whether or not a family would be capable of using telehealth to do something that's fairly straightforward"

"... the kids with special needs, probably more likely to offer a telehealth, because the parents are... pretty well trained in managing the device. And, between the two of us, we can problem solve and see if there's a problem. I trust the parents to say, "Oh, he is getting some pressure. I'm going to come into the clinic instead of a telehealth."

"I certainly offer telehealth to the families who are on their second, third, fourth, fifth set of orthosis. They know exactly what they're doing. They know what the red marks look like. They know how things should fit, and look, and feel and work."

Dillon et al. 2024

Future-proofing telehealth

There are some key issues that will impact the **sustainability** of a well-regulated and high-quality telehealth service into the future.

EDUCATION

Practitioners who were knowledgeable about telehealth were more engaged and better equipped to solve some of the challenges.

There are opportunities for universities, clinical facilities, and professional associations to provide education that helps build capability to provide a high-quality telehealth service.

FUNDING

There are conflicting views about how telehealth services should be reimbursed.

As a profession, we need an agreed position to effectively advocate for the reimbursement required to support a well-regulated, sustainable, and high-quality telehealth service into the future.

REGULATION

Current regulation is complex given the different federal, state, and profession-specific associations involved.

A right-touch approach is required; one that empowers practitioners and patients to continue to make thoughtful decisions about when telehealth can be used to provide safe and effective O&P care.

More information

While we hope that this guide has served as a useful introduction to telehealth for the O&P profession, we appreciate that you may want **more information**.

SCOPE OF PRACTICE

If you have questions about telehealth and your scope of practice or regulation, we encourage you to connect with your professional association.

EDUCATION

We encourage practitioners to undertake explicit telehealth education and training available through telehealth software providers, or local universities.

COMMUNITY OF PRACTICE

There are opportunities to build a community of practice with other clinical facilities to solve some of the practice-related challenges like scheduling or medical record keeping.

RESEARCH

If you'd like to know more about the research that underpins this guide, we encourage you to look to the peer reviewed articles listed in the inside cover.

LA TROBE UNIVERSITY

University of South Australia
