# Supplementary material for: Exploration of the barriers and facilitators influencing use of telehealth for orthotic/prosthetic services in the United States of America: An orthotist/prosthetists perspective
Source: PLoS One. 2024 Oct 24;19(10):e0309194. doi: 10.1371/journal.pone.0309194 (PMC11500877; doi:10.1371/journal.pone.0309194)
Supplement: S3 File — (DOCX) [file pone.0309194.s004.docx]

Inclusivity in global research

PLOS’ policy on inclusivity in global research aims to improve transparency in the reporting of research performed outside of researchers’ own country or community and ensures that PLOS publications reporting global research adhere to high standards for research ethics and authorship. Authors of relevant research articles may be asked to complete the questionnaire below, which outlines ethical, cultural, and scientific considerations specific to inclusivity in global research. This questionnaire may be requested when researchers have travelled to a different country to conduct research, if research uses samples collected in another country, research with Indigenous populations or their lands, or if research is on cultural artefacts. Researchers travelling to another country solely to use laboratory equipment will not normally be required to complete the questionnaire. However, the questionnaire can be requested at the journal’s discretion for any submission – if you have been requested to complete this questionnaire by the PLOS journal you submitted to, please do so.

Please complete the questionnaire below and include this as a Supporting Information file with your manuscript. Note that if your paper is accepted for publication, this checklist will be published with your article in the supporting information files. Please ensure that you reference the checklist in the main body of your manuscript. We suggest adding a subsection ‘Inclusivity in global research’ to your Methods section and adding the following sentence: “Additional information regarding the ethical, cultural, and scientific considerations specific to inclusivity in global research is included in the Supporting Information (SX Checklist)”

The questions have been designed to be applicable to a wide range of study types, and there are subsections for both human subjects research and non-human subjects research. If any of the questions are not relevant to your research please mark them as “N/A” as appropriate.

**Ethical considerations, permits and authorship**

*This section is applicable to all research types.*

Provide details as to who granted permissions and/or consent for the study to take place in the Methods section of your manuscript. This should include the names of **all** ethics boards, governmental organizations, community leaders or other bodies that provided approval for the study. If individuals provided approval refer to these people by their role or title but do not list their name(s).

Reported on page number: Page 2, Ln 44-45

If there were any deviations from the study protocol after approval was obtained please provide details of these changes in the Methods section of your manuscript.
Did this study involve local collaborators that are residents of the country where the research was conducted or members of the community studied? If you do not have any authors from said communities, please provide an explanation for this below.

No, there were no deviations from the approved study.

During the course of the study, Dr Emily Ridgewell lived and worked in the United States of America. Dr Ridgewell was employed by La Trobe University (Melbourne Australia) which was the administering institution for this grant and the research undertaken.

Everyone listed as an author should meet PLOS’ criteria for authorship and all individuals who meet these criteria should be included in the author byline, rather than the acknowledgements. For further information please see the journal’s Authorship Policy.

All authors listed on the manuscript meet the requirements for authorship.

**Human subjects research (e.g. health research, medical research, cross-cultural psychology)**

Did you obtain written informed consent from a representative of the local community or region before the research took place? How did you establish who speaks for the community? Details of written informed consent obtained from study participants should be reported separately in the Methods section of your manuscript.

Details about informed consent have been reported in the Methods section of the manscript, including the provision of a Participant Information Statement (Pg 3, Ln 62) and the request for verbal consent from participants (Pg 4, Ln 81-84). The request for verbal consent following a structured approach that included participants describing the purpose of the study in their own words, confirming their eligibility, and asking any questions about the study. In this way, the researchers could be confident that participants knew about the purpose of the study, the procedures, and were able to provide verbal consent. The process for obtaining consent was approved the the ethics committes responsible for overseeing this work.

How did members of the local community provide input on the aims of the research investigation, its methodology, and its anticipated outcome(s)? When engaging with the local community, how did you ensure that the informed consent documents and other materials could be understood by local stakeholders?

The professional association that funded this work was based in the United States of America and included representatives from the profession (e.g., clinicians, researchers). In panel assembled to review the grant application included individuals with the skills and capability to assess the merit of the proposed method and its applicability for US-based O&P practitioners; including the ability of practitioners to review the study documents in English. As a piece of qualitative research, we were able to adapt the lines of questioning over time as our understanding of the local context grew, and test our understanding. We also worked with an expert advisory group who provided input into the telehealth guide that was included as a supplement to ensure the content was appropriate for use in a US setting. That advisory group helped ensure the language and terminology was appropriate to the USA setting.

Will the findings of the research be made available in an understandable format to stakeholders in the community where the study was conducted (e.g. via a presentation, summary report, copies of publications, etc.)? Please provide details of how this will be achieved.

Yes, the research will be made available in a peer reviewed journal article which is appropriate for Masters-degree trained health professionals in the USA. We will also explore other avenues for dissemination through social media, conference presentations etc as opportunities arise.

**Non-human subjects research using specimens/ animals collected as part of the study, or those housed in archival collections. Examples include archaeology, paleontology, botany and zoology.**

Did the permission you obtained from a local authority to perform the study include an agreement on access to outputs and benefit sharing? This may include procedures to enable fair distribution of the benefits and resources arising from the research performed. Please include any details of Prior Informed Consent and Benefit Sharing Agreements obtained. These may be required by field-specific regulations, for example the Convention on Biological Diversity (CBD) and the associated Nagoya Protocol.

Not applicable

If the material used in your study was imported, please A) provide the year it was imported and B) indicate whether permits were obtained to import/export the materials used, C) provide details of any permits obtained. If this information is not available, please indicate this.

Not applicable

If you used archival specimens, please state how the material used in your study was acquired by the institute it is held in and provide details of any permits obtained for the original excavations/ sample collection. If this information is not available, please indicate this.

Not applicable

How was the potential cultural significance of the materials collected in your study to local communities considered in your research design? Were Indigenous peoples and/or local researchers and institutions involved with archaeological excavations / collection of specimens? If so, please provide a description of their involvement.

Not applicable

If your manuscript includes photographs of human remains please indicate whether authors obtained permission from descendants or affiliated cultural communities to do so.

Not applicable
